# Implementation of the Xpert MTB/RIF assay for tuberculosis in Mongolia: a qualitative exploration of barriers and enablers

**DOI:** 10.7717/peerj.3567

**Published:** 2017-07-14

**Authors:** Nicole L. Rendell, Solongo Bekhbat, Gantungalag Ganbaatar, Munkhjargal Dorjravdan, Madhukar Pai, Claudia C. Dobler

**Affiliations:** 1 National Tuberculosis Program Mongolia, Ulaanbaatar, Mongolia; 2 Mongolian Anti-Tuberculosis Association, Ulaanbaatar, Mongolia; 3 McGill International TB Centre, McGill University, Quebec, Canada; 4 Woolcock Institute of Medical Research, University of Sydney, New South Wales, Australia; 5 South Western Sydney Clinical School, University of New South Wales, New South Wales, Australia

**Keywords:** Tuberculosis, Xpert MTB/RIF, Mongolia

## Abstract

**Objective:**

The aim of our study was to identify barriers and enablers to implementation of the Xpert MTB/RIF test within Mongolia’s National Tuberculosis Program.

**Methods:**

Twenty-four****semi-structured interviews were conducted between June and September 2015 with laboratory staff and tuberculosis physicians in Mongolia’s capital Ulaanbaatar and regional towns where Xpert MTB/RIF testing had been implemented. Interviews were recorded, transcribed, translated and analysed thematically using NVIVO qualitative analysis software.

**Results:**

Eight laboratory staff (five from the National Tuberculosis Reference Laboratory in Ulaanbaatar and three from provincial laboratories) and sixteen tuberculosis physicians (five from the Mongolian National Center for Communicable Diseases in Ulaanbaatar, four from district tuberculosis clinics in Ulaanbaatar and seven from provincial tuberculosis clinics) were interviewed. Major barriers to Xpert MTB/RIF implementation identified were: lack of awareness of program guidelines; inadequate staffing arrangements; problems with cartridge supply management; lack of local repair options for the Xpert machines; lack of regular formal training; paper based system; delayed treatment initiation due to consensus meeting and poor sample quality. Enablers to Xpert MTB/RIF implementation included availability of guidelines in the local language; provision of extra laboratory staff, shift working arrangements and additional modules; capacity for troubleshooting internally; access to experts; opportunities for peer learning; common understanding of diagnostic algorithms and decentralised testing.

**Conclusion:**

Our study identified a number of barriers and enablers to implementation of Xpert MTB/RIF in the Mongolian National Tuberculosis Program. Lessons learned from this study can help to facilitate implementation of Xpert MTB/RIF in other Mongolian locations as well as other low-and middle-income countries.

## Introduction

In December 2010, the World Health Organization (WHO) formally endorsed use of Xpert MTB/RIF as a new diagnostic tool for active tuberculosis (TB) disease (World Health Organization, 2016a). Since then, use of Xpert MTB/RIF has increased dramatically. Between 2010 and 2015, under concessional pricing arrangements, 4,672 GeneXpert machines had been procured in 122 of the 145 eligible countries, including Mongolia (World Health Organization, 2016b).

Mongolia, a country with a TB burden of 428 incident cases per 100,000 population in 2015 (95% confidence interval 220–703) (World Health Organization, 2017), has been a relatively late adopter of the Xpert MTB/RIF assay. In Mongolia, the rate of concomitant HIV and TB is low at 0.34 incident cases per 100,000 population (95% CI [0.26–0.44]), but the country has a relatively high burden of multi-drug resistant (MDR) and rifampicin resistant (RR) TB cases representing 2.2% (95% CI [1.1–3.3]) of new cases and 33% (95% CI [29–38]) of previously treated cases (World Health Organization, 2017). Resistance against all first-line drugs tested is approximately 60% among sputum smear-positive patients in whom standard first-line TB treatment failed, suggesting successful transmission (versus new mutation) of these highly resistant strains in the community (Dobler et al., 2015). At the time of the first introduction of MTB/RIF in Mongolia at the end of 2013, other countries had already analysed their experiences with implementation of Xpert MTB/RIF (Creswell et al., 2014). It was, however, not until 2014 that the WHO published a ‘How-To’ Xpert MTB/RIF implementation manual (World Health Organization, 2016c). It is therefore of interest to explore the specific challenges with Xpert MTB/RIF implementation in Mongolia.

The National Tuberculosis Reference Laboratory (NTRL) in Ulaanbaatar was the first laboratory in Mongolia to implement Xpert MTB/RIF testing in November 2013. By the end of 2014, there were three GeneXpert machines being used in Mongolia—at the NTRL in Ulaanbaatar, the Hospital of Darkhan-uul, in the northern Darkhan-uul province, near the Russian border, (since June 2014) and the Regional Diagnostic and Treatment Center of Dornod, in Dornod province, Mongolia’s easternmost province, bordering Russia and China (since March 2014). The funding for these machines was supplied by the Global Fund through a series of projects. In 2013, 310 patient samples were tested using Xpert MTB/RIF. This had increased to 3,289 in 2014, 3,802 in 2015 and 3,991 in 2016 (Table 1).

Diagnostic tools such as Xpert MTB/RIF must be considered in the context of their organisational environment to understand the value of their technical benefits in real terms. Such benefits include the relatively short time frame to determine a result (2 h) and ease of use compared to other diagnostic methods. Understanding the operational issues associated with implementation of the Xpert MTB/RIF test ensures these technical benefits can be maximised.

**Table 1:** Number of patient specimens tested using Xpert MTB/RIF, by laboratory, in Mongolia, 2013–2014.

Laboratory location	Commencement	Number of patient specimens^a^ tested			
		2013	2014	2015	2016
Ulaanbaatar	November 2013	310	3,022	3,493	3,588
Dornod	March 2014	–	130	114	162
Darkhan-uul	June 2014	–	137	195	241

**Notes.**

a Specimens include all pulmonary and extra-pulmonary specimens.

In recent years a number of studies have assessed the performance of the Xpert MTB/RIF assay, specifically, the accuracy and cost effectiveness of Xpert MTB/RIF have been well documented (World Health Organization, 2016d; Arm et al., 2011; Nhu et al., 2013; Huh et al., 2014; Menzies et al., 2012; Vassall et al., 2011). Only few research studies, however, have investigated implementation issues from an organisational perspective (Creswell et al., 2014; Engel et al., 2015a; Durovni et al., 2014; Raizada et al., 2014; De Camargo Jr et al., 2015; Albert et al., 2016), two of which included qualitative tools designed specifically for the study to gather information on user experiences and challenges related to Xpert MTB/RIF implementation (Creswell et al., 2014; De Camargo Jr et al., 2015). Some of the identified challenges associated with Xpert MTB/RIF implementation in low-and middle income countries include lack of standardised guidance, piecemeal implementation of training, quality assurance, planning processes and equipment servicing and maintenance, issues with continuous power supply and difficulties recording and reporting test results using new technology (Creswell et al., 2014; De Camargo Jr et al., 2015; Albert et al., 2016).

The aim of our study was to identify and understand system and context specific factors within Mongolia’s National Tuberculosis Program (NTP) that are barriers or enablers to implementing the Xpert MTB/RIF test from the perspective of NTP staff.

## Methods

### Study design, setting and participants

We conducted semi-structured interviews with laboratory staff and TB physicians using an inductive-deductive approach. This approach was chosen because we had knowledge about possible barriers and enablers to GeneXpert implementation from studies in other settings (Creswell et al., 2014; Engel et al., 2015a; Durovni et al., 2014; Raizada et al., 2014), while we knew little about contextual factors impacting on Xpert MTB/RIF implementation in Mongolia. We developed semi-structured interview guides (see Appendix A) to collect a wide range of information on participants’ experiences and views related to Xpert MTB/RIF use and implementation in Mongolia’s NTP. The interview guide was designed based on a literature review of barriers and enablers of Xpert MTB/RIF implementation. These implementation factors were organised into major themes that formed the structure of the interview guide for laboratory staff: guidelines and organisational structures; equipment; training; communication systems; and diagnostic algorithms, case finding for Xpert MTB/RIF, clinical management. A separate interview guide was designed specifically for TB physicians and focused on clinical issues. Open questions were added to the interview guide to explore possible themes that had not been identified from the literature. Two of the researchers (GG and MD) conducted the interviews. Both interviewers were trained in the study protocol and interview technique.

Participants included laboratory staff (doctors, technicians and administrative officers) who were using the Xpert MTB/RIF diagnostic test and TB physicians who were working either at the Mongolian National Center for Communicable Diseases (NCCD) or in any of the district TB clinics in Mongolia’s capital Ulaanbaatar, or in the provinces of Darkhan-Uul or Dornod.

Potential participants were approached by one of the researchers (GG or MD) and interviewed once informed consent was obtained. Because there were only few laboratory staff who were using the Xpert MTB/RIF diagnostic test, all of them were invited to participate in the study. Interviews with TB physicians were continued until saturation of data was apparent, that is until no new themes emerged (Patton, 1990). All interviews were conducted between July and September 2015.

Participants in Ulaanbaatar were interviewed in person where possible, or alternatively over the phone, and participants based outside of Ulaanbaatar were interviewed over the phone. The interviews were conducted and transcribed in Mongolian. The transcripts were then translated into English by one of the researchers (SB). The English transcripts were verified by another bi-lingual member of the research team (MD) to ensure the English version was clear and the participants’ views were adequately represented.

### Analysis

In order to determine whether participants would interpret interview questions as intended and whether the order of questions may influence responses, we conducted three pretesting interviews (with two laboratory staff and one TB physician). Pretesting highlighted questions that were poorly understood by respondents. It also showed that some questions were perceived by respondents to be duplicate questions. We edited the interview guides accordingly by reordering, rewording or deleting questions. In addition, pretesting showed that explanatory information provided by the interviewer varied considerably between interviews. In response to this observation, a written introduction explaining the purpose and benefit of the study was added to the interview instrument for the interviewers to read aloud before the interview commenced. This ensured consistent explanatory information was given to all participants prior to the commencement of the interview. The revised interview guides were used for all future interviews. The pretest interviews were not included in the final analysis.

The first (NR) and last author (CCD) independently reviewed transcripts from the first five interviews to identify key themes and subthemes (i.e., patterns within the narrative data), then developed a coding scheme through discussion and consensus. This framework was systematically applied to code themes in subsequent interview transcripts. We reviewed the framework after an additional ten interviews, updating it to reflect newly revealed themes and subthemes that were not apparent in earlier interviews. Coding was completed using the software package QSR NVivo version 10 (QSR International Pty Ltd, Doncaster, Victoria, Australia). The English version of each interview transcript was imported into NVivo and then systematically reviewed and coded for common themes and subthemes.

The study results are reported in accordance with the Consolidated Criteria for Reporting Qualitative Research (COREQ) checklist (Tong, Sainsbury & Craig, 2007).

### Ethics

The study was approved by the Scientific Council of the Mongolian NCCD.

## Results

We contacted 24 NTP staff (8 laboratory staff and 16 TB physicians), all of whom agreed to be interviewed (i.e., a 100% response rate). Five laboratory staff members were located at the NTRL in Ulaanbaatar and three in provincial laboratories. Of the 16 TB physicians, five worked at the NCCD, four worked in any of the district TB clinics in Ulaanbaatar and seven worked in the provinces of Darkhan-Uul or Dornod.

Participants identified a range of barriers and enablers associated with implementation of Xpert MTB/RIF testing in Mongolia based on their experiences. These are summarised in Table 2, and described below according to the following themes: (1) guidelines and organisational structures, (2) equipment, (3) training, (4) communication systems, (5) diagnostic algorithms, case finding for Xpert MTB/RIF and clinical management. Laboratory staff participants were interviewed using guiding questions for each theme. For TB physicians, the guiding questions focussed on the last theme: diagnostic algorithms, case finding for Xpert MTB/RIF and clinical management.

**Table 2:** Barriers and enablers of Xpert MTB/RIF implementation, 2015.

Barriers	Enablers
**Guidelines and organisational structures**	
• Poor awareness of program guidelines—staff members were not always aware that guidelines existed and how they could be accessed • Inadequate staffing arrangements—laboratory staff participants indicated an increase in their workload without a change in staffing arrangements to accommodate the increased workload.	• Clear guidelines in local language—in situations where participants were aware of guidelines in Mongolian, they were considered valuable guidance for working with Xpert MTB/RIF • Extra staff in NTRL, shift working arrangements and/or an increase in the number of modules—these arrangements would have assisted staff in meeting the increased demands that resulted from the introduction of Xpert MTB/RIF.
**Equipment**	
• Poor supply chain management of cartridges (stock-outs)—this happened on one occasion and meant Xpert MTB/RIF testing ceased. • Absence of local repair options—difficulties were reported for arranging repairs when required because of limited availability of trained mechanics to repair Genexpert machines in Mongolia.	• Capacity for troubleshooting internally—there where some situations where the participants were able to determine the cause of machine faults and resolve them using a locally sourced solution. This meant little interruption to the work flow.
**Training**	
• Inconsistent formal training options—some laboratory staff participated in formal training courses, others learned on the job through instruction by trained colleagues or superiors.	• Access to experts—some participants had direct access to visiting international technical experts • Peer learning—all staff recognised the value of learning from their colleagues.
**Communication Systems**	
• Paper based system—storing patient information on paper forms through the laboratory workflow meant results were communicated inefficiently through a paper based system and administrative reporting required a manual input of information.	
**Diagnostic algorithms, case finding for Xpert MTB/RIF and clinical management**	
• Treatment initiation in MDR-TB delayed until after consensus meeting—some participants reported a delay in initiating MDR-TB treatment because of the procedural requirement to determine the MDR-TB treatment plan at a weekly meeting in Ulaanbaatar. • Poor sample quality—this was the most commonly reported error experienced by study participants.	• Common understanding of indications for Xpert MTB/RIF testing (diagnostic algorithms)—all participants reported an awareness of the indications for Xpert MTB/RIF use in Mongolia • Testing availability in provincial centres (decentralised)—participants in provincial clinics value the accessibility of a tool to diagnose MDR-TB locally.

### Guidelines and organisational structures

#### Barriers

***Poor awareness of program guidelines:*** Around half of all participants stated that they were not aware of any written guidelines to support the use of Xpert MTB/RIF *(authors’ comment: the Mongolian NTP issued guidelines for all NTP staff, which included information on the use of Xpert MTB/RIF in December 2014).* Instead, many participants referred to training arrangements when asked about NTP guidelines. One participant thought that the English manual for the operation of the Xpert machine was equivalent to the NTP guidelines available to staff. The guidelines did not appear to be clear enough about which samples (type of body fluid/tissue) could be used for testing with Xpert MTB/RIF, in particular if testing of samples other than sputum, e.g., pleural and cerebrospinal fluid, was allowed. This resulted in inconsistent practices regarding Xpert MTB/RIF testing of non-sputum samples (*authors’comment: According to the Mongolian NTP guidelines 2014 the following specimens can be used for Xpert MTB/RIF testing: sputum, urine, stool, pleural fluid, ascites, gastric lavage, and surgical tissue samples, although the protocol for collecting and transporting specimens collected for testing was not included in the guidelines at the time of the study*). One laboratory participant suggested that patients are being inappropriately referred for Xpert MTB/RIF testing (inconsistent with guideline recommendations, summarised in Table 3), which was potentially driving excess demand.

**Table 3:** Summary of indications for clinicians to prescribe the Xpert MTB/RIF test, Mongolian National Tuberculosis Program Guidelines, December 2014.

Indication	Additional detail
All smear negative pulmonary TB cases	
Patient with presumed pulmonary TB diagnosed with HIV/AIDS	
Patients with presumed MDR-TB	• Smear positive at the 2nd (3rd) and 5th month of TB treatment with category I and II • Smear positive after interruption of TB treatment category I and II • Relapse (after TB treatment category I and II) • Indefinite previous treatment regimen or smear negative • Smear positive after being smear negative during treatment initiation • Identification of TB case from contact investigation of a DR-TB case.
Patients with presumed XDR-TB	• Used category II drugs for 2 or more months • Culture positive at the 3rd month of MDR-TB treatment • Not converted to negative at the end of intensive treatment phase of MDR-TB • Smear positive again by bacteriological analysis after conversion to negative during continuous treatment phase of MDR-TB • MDR-TB case defined to be resistance to fluoroquinolone group or second line injectable TB drugs • Close contacts of XDR-TB case.
All smear positive new cases aged 15–34 years old (this guideline is yet to be implemented)	

**Notes.**

These indications are a summary adapted from treatment algorithms and other guidance outlined within the Mongolian National Tuberculosis Program Guidelines (Ministry of Health Mongolia, 2014).

***Inadequate staffing arrangements:*** Participants identified that their workload had increased in response to the introduction of the Xpert MTB/RIF testing into their laboratories, and inadequate staffing was mentioned as a problem by staff of the NTRL in Ulaanbaatar.

#### Enablers

***Clear guidelines in local language:*** Participants, who were aware of the NTP guidelines, found the guidelines useful for working with Xpert MTB/RIF and they understood how to apply the guidelines in practice.

***Extra staff, shift working arrangements, increase of Xpert MTB/RIF modules:*** Staff of the regional laboratories felt that while there was an initial increase in their workload, their staffing arrangements and laboratories were able to accommodate the change. Staff of the NTRL in Ulaanbaatar suggested a range of options to improve their ability to meet the demand. These included additional staff, a staff member allocated only to Xpert MTB/RIF, shift arrangements, installing more GeneXpert machines or a new GeneXpert machine with a greater number of modules (in order to perform more tests at the same time).

### Equipment

#### Barriers

***Poor supply chain management of cartridges (stock-outs):*** Since implementation of Xpert MTB/RIF testing, the only time the test had been unavailable for about a month was due to a cartridge supply issue that affected all the laboratories that offered Xpert MTB/RIF testing. Other than this occasion, cartridge supply appeared to be well managed (sufficient number of cartridges available to meet demand without cartridge expiration issues due to oversupply).

***Absence of local repair options:*** Mongolia’s harsh climate and low population density generally did not appear to have affected transportation of cartridge supplies or equipment. Although, one of the modules in one of the regional machines had broken on arrival and there were difficulties arranging its repair. The lack of expertise to repair GeneXpert machines in the provinces was identified as a problem. This was in contrast to other laboratory equipment, which could be repaired by the available engineers.

#### Enabler

***Capacity for troubleshooting internally:*** Other temporary problems with the GeneXpert machine have occurred without major interruption to the workflow because local staff were able to remedy them easily. For example, one laboratory participant mentioned that the laboratory had bought an uninterruptible power supply unit to manage issues with the power supply. Another laboratory participant mentioned the need for more basic office equipment to cater for the introduction of the new equipment.

### Training

#### Barrier

***Inconsistent formal training options:*** The training that laboratory staff had received on the operation of Xpert MTB/RIF varied widely. Some laboratory staff participated in formal training courses, others learned on the job through instruction by trained colleagues or superiors. Two members of laboratory staff based in Ulaanbaatar participated in an online training course organised by the supplier of the GeneXpert machines. Components of different trainings included operation and maintenance of GeneXpert, but also information on indications for GeneXpert use in Mongolia.

When asked about the value of the training received, most laboratory staff felt that it was sufficient to meet their job requirements. While staff in Ulaanbaatar were satisfied that the training they had received equipped them to use Xpert MTB/RIF testing, regional laboratory staff were keen to receive further training.

There were also differences in the training opportunities provided for other TB diagnostic methods, primarily smear testing. Laboratory staff were aware of scheduled annual trainings for smear testing, but were unsure about any future and/or regular ongoing training opportunities for Xpert MTB/RIF testing.

#### Enablers

***Access to experts:*** Access to external technical experts to support implementation of Xpert MTB/RIF differed for staff based in regional clinics and those based in Ulaanbaatar. Regional laboratory staff received guidance from NTRL staff, who received guidance directly from international experts on operations and troubleshooting of the machines.

***Peer learning:*** All staff recognised the value of learning from their peers.

### Communication systems

#### Barrier

***Paper based system:*** Results of the Xpert MTB/RIF test were communicated to TB clinics using paper forms that were delivered using one or more of the following options: (1) patients collected the paper form directly from the laboratory and took it to the doctor at the TB clinic, (2) doctors were contacted over the phone and informed about the results ahead of receiving the paper form, and (3) doctors and/or nurses collected the results on paper directly from the laboratory. Paper reports were associated with delays of 2 days on average to receive the results following a request being sent to the laboratory. A few TB physicians suggested the delay was longer, up to a week, and one TB physician even suggested that the delay between requesting Xpert MTB/RIF testing to receiving the result was up to 2 weeks. Urgent requests were prioritised.

Administrative reports were generated weekly on a computer, based on the information in the paper forms that was manually input into a computer file. Despite having regular administrative reporting of the results to facilitate a quality control mechanism, participants did not value the reporting system in this way. It is unclear based on the interview data how the information contained in the reports could be used to prompt action to improve processes.

### Diagnostic algorithms, case finding for Xpert MTB/RIF and clinical management

#### Barriers

***Treatment initiation in MDR-TB delayed until after consensus meeting***: Treatment for drug sensitive cases (who tested positive for TB on GeneXpert, but negative for rifampicin resistance) was usually started immediately after physicians received the results. TB physicians described that the delay to initiating treatment for presumptive MDR-TB (following a positive result for rifampicin resistance), as well as the specifics of the MDR-TB treatment regimen depended on the timing of the weekly MDR-TB meeting (held at the central NTP office in Ulaanbaatar, to discuss each case of MDR-TB in Mongolia and decide on the best approach for the patient’s treatment). Some participants reported that there was no delay in commencing treatment following a positive test for rifampicin resistance. Others reported that it took 1–2 weeks to initiate treatment because the treatment could not commence until the treatment plan had been agreed upon at the MDR-TB meeting.

***Poor sample quality:*** ‘Error results’ from Xpert MTB/RIF testing (i.e., notification of an error is displayed in the Check Status screen of the GeneXpert machine) were reported to occur occasionally. In these instances, the test was always redone and some participants reported that error results triggered machine maintenance as well. The quality of the sample (e.g., when patients did not collect sputum correctly) was reported as the most common reason for error results experienced by participants when using Xpert MTB/RIF testing. Some laboratory staff participants also reported seeking additional training and/or assistance to minimise error results.

#### Enablers

***Common understanding of indications for Xpert MTB/RIF testing****:* Both laboratory staff and TB physicians were aware of the approved indications for Xpert MTB/RIF use in Mongolia (Table 3), including testing in patients with possible MDR-TB (patients with TB relapse, positive sputum smear result at the 3rd and 5th month of treatment and screening of MDR-TB close contacts), as well as testing for confirmation of TB (smear negative cases with abnormal chest X-ray and very ill patients with uncertain diagnosis).

There was variation in the details of each participant’s response regarding indications for GeneXpert use, but broadly the indications as outlined above were consistent among participants and consistent with Mongolian guidelines (see Table 3).

***Testing availability in provincial centres (decentralised):*** Participants from the provinces pointed out that while Xpert MTB/RIF testing is available in regional areas of Mongolia, samples need to be sent to the central reference laboratory in Ulaanbaatar for further drug susceptibility testing from culture. They did, however, still value access to Xpert MTB/RIF testing, because it meant that a rapid diagnosis of drug resistant TB was possible in the regional areas when previously, it could only be done in Ulaanbaatar.

Most physicians had not experienced difficulties starting their patients on MDR-TB treatment following a diagnosis of rifampicin resistance. However, those that did reported that the reason they could not commence treatment was because of patient related factors rather than program related issues.

### Suggestions for future Xpert MTB/RIF implementation and scale up

All study participants viewed the new diagnostic technology positively and appreciated the time saving benefits unique to the Xpert MTB/RIF test. When asked about what future changes they would like to see to implementation of Xpert MTB/RIF testing in Mongolia, participants highlighted the need for change in the following three areas:

• Increase in the number of available sites for Xpert MTB/RIF testing (upscaling to more districts of Ulaanbaatar and more provinces in Mongolia).
• Use of Xpert MTB/RIF to test for drug resistance on all smear positive patients before starting treatment.
• Increase in the number of machines at existing sites.

## Discussion

This study highlighted a range of potential factors within the Mongolian NTP that served as a barrier or enabler to the implementation of Xpert MTB/RIF testing. Since the implementation of Xpert MTB/RIF testing in Mongolia, according to NTP data, the number of notified cases detected in Ulaanbaatar, Darkhan and Dornod has increased from 2,783 cases in 2012 to 3,029 cases in 2015. While all study participants appreciated the benefits of the new diagnostic technology and were supportive of its implementation, all participants were able to identify areas where integration into the existing program could be improved. Potential barriers included lack of awareness of program guidelines; inadequate staffing arrangements; problems with cartridge supply management; lack of local repair options for the Xpert machines; lack of regular formal training; paper based system; delayed treatment initiation due to consensus meeting and poor sample quality. Enablers included availability of guidelines in the local language; provision of extra laboratory staff, shift working arrangements and additional modules; capacity for troubleshooting internally; access to experts; opportunities for peer learning; common understanding of diagnostic algorithms and decentralised testing. Lessons learned from this study can inform the implementation and upscaling of GeneXpert in other settings in Mongolia and in other low-and middle income countries.

Policies and guidelines as well as training opportunities provided by an organisation facilitate the uptake of any new technology (World Health Organization, 2016c). In our study, those that knew about the NTP’s guidelines for the use of GeneXpert found them to be invaluable. However, only around half of study participants were aware of any guidelines relating to GeneXpert use and relied on random opportunities for training and/or visits from national and international experts to advise them on the use of Xpert MTB/RIF. Adherence to program guidelines, which should incorporate Xpert MTB/RIF testing into the national diagnostic strategy and algorithms (World Health Organization, 2016c), is important for sustainable implementation of Xpert MTB/RIF in a local context. Diagnostic algorithms and other policies outlined in program guidelines are essential to realise and maximise the potential of new technologies in practice (McNerney et al., 2015; Kirwan, Cárdenas & Gilman, 2012; Giang et al., 2015).

Only staff from NTRL, but not from the regional laboratories, had concerns about their capacity to meet the demand for Xpert MTB/RIF testing. This is most likely because the regular requests for the population that the NTRL laboratory covered went above their pre-existing surge capacity. In contrast, the regional laboratories only felt under pressure initially, while they were still learning how to use the technology. A feasibility study conducted in India also found that in a decentralised setting, there were minimal infrastructure modifications and human resource concerns following the implementation of Xpert MTB/RIF testing (Raizada et al., 2014). The lower population density in settings covered by existing laboratories in the decentralised and regional areas, potentially allowed the introduction of new diagnostic technology using the pre-existing surge capacity of the laboratory workforce.

Issues with the infrastructure supporting Xpert MTB/RIF testing were observed by many laboratory staff and focused on the one month where cartridge supply was an issue, and to a lesser extent, access to repairs. In our study the demand for cartridges was met without expiration issues due to high turnover of cartridges, except for one month when the cartridge supply was exhausted. No Xpert MTB/RIF testing could be undertaken during this month. Previous studies investigating Xpert MTB/RIF implementation have highlighted the importance of program levers to assist in the management of cartridge supply (Creswell et al., 2014; Durovni et al., 2014). They have suggested measures such as staggered cartridge shipments and design of diagnostic algorithms that combine Xpert with other methods to increase yield (Creswell et al., 2014; Durovni et al., 2014). A Brazilian study assessing the pilot implementation of Xpert MTB/RIF calculated that 10% of Xpert MTB/RIF equipment needed replacement during the eight month pilot study, yet spare parts were not immediately available (Durovni et al., 2014). Although our study did not quantify faulty equipment, a similar experience of limited access to repairs was observed by laboratory staff. The authors of the Brazilian study suggested that both cartridge supply and maintenance should be negotiated with manufacturers (Durovni et al., 2014).

Communication of Xpert MTB/RIF results occurred through different methods that were paper based and reliant on the accuracy of the contact information written on the Xpert MTB/RIF request form. Interestingly, no participant would have preferred computer based record keeping or an online system, even though administrative reports were generated weekly on a computer, based on the information in the paper forms, manually input into the computer files. Regular reporting in this way is inefficient and did not appear to facilitate a quality control mechanism that would be of value to the participants. A study undertaken in two Brazilian cities found that the implementation of concomitant IT technology to support Xpert MTB/RIF integration into organisational workflows led to delays in physicians receiving laboratory results (De Camargo Jr et al., 2015). In that study, difficulties with the online system were thought to be a result of poor knowledge on how to use the equipment and networks effectively, low availability of online systems and available systems not interacting with each other leading to repeated input of data (De Camargo Jr et al., 2015). Given the limited availability and uptake of electronic databases and online systems within Mongolia’s NTP, it is reasonable to assume that Mongolian NTP staff may have concerns about upscaling IT technology that echo the experience outlined in the Brazilian study (De Camargo Jr et al., 2015). However, IT solutions enable more efficient record keeping internally and capacity for connectivity externally to pool surveillance data that can benefit TB control efforts within the region (Andre et al., 2016).

Several studies have highlighted issues with sample quantity and quality for GeneXpert testing (Creswell et al., 2014; Durovni et al., 2014; Raizada et al., 2014). Our findings are consistent with these studies—participants suggested the most common reason for experiencing an error message was related to the sample’s quantity and/or quality. Specimen transportation systems are in place at the three levels of the health system—community, district/provincial and central. All health care workers with responsibility for collecting specimens receive training on the collection and effective transportation of specimens. When collecting specimens, patients are provided with verbal and written instructions. Participants also reported conflicting information on the type of specimen that could be used for Xpert MTB/RIF testing, indicating the need for better education and training in this area.

The rapid testing feature of Xpert MTB/RIF (results available in 2 h) means that the technology is poised for point of care (POC) testing. Our study supports the findings of studies on POC testing that found that simply having the rapid test technology available does not guarantee a natural fit into the program environment to enable POC testing (Engel et al., 2015a; Cowan et al., 2015; Pai et al., 2012; Clouse et al., 2012). Detailed recommendations on how to facilitate integration of GeneXpert with clinical care have been made (Dominique et al., 2015). Two of these recommendations, standardised training and establishment of automated processes for prescriptions (Dominique et al., 2015), would address important barriers identified in our study.

In our study, processes that followed a diagnosis of MDR-TB contributed to time delays until treatment initiation despite the rapid availability of Xpert MTB/RIF results. An Indian study on a POC testing program found that diagnostic delays, such as those observed in our study, undermined the full potential of rapid tests (Engel et al., 2015b). However, two trials suggest that the benefit of prompt treatment initiation gained using Xpert MTB/RIF may not translate into improved patient outcomes. One randomised controlled trial found that nurses in African primary care clinics had the capacity to accurately administer Xpert MTB/RIF at the clinic, which resulted in more patients starting same-day treatment, more culture-positive patients starting therapy and a shorter time to treatment initiation compared to the use of sputum smear microscopy (Theron et al., 2014). However, the trial also found that these benefits did not translate into lower tuberculosis related morbidity (Theron et al., 2014). The other trial, a South African cluster-randomised trial, found that there was no reduction in mortality at six months when using Xpert MTB/RIF compared to smear microscopy (Churchyard et al., 2015). In Mongolia, Xpert MTB/RIF is currently not implemented as a POC test, as the laboratories performing the testing are not directly integrated with the TB clinics. Improving integration of GeneXpert with clinical care by addressing some of the issues outlined above could potentially increase the value of GeneXpert testing.

More broadly, funding arrangements and health system functioning have also been identified in the literature to have an important role in the implementation of new diagnostic technologies (Engel et al., 2016). However, these concepts were outside the scope of this study because we focused on the experiences of operational staff.

A limitation of our study was that interview questions and answers had to be translated from English into Mongolian and from Mongolian into English respectively, possibly resulting in loss or misinterpretation of information. To reduce these risks, we pretested the interview guides and adjusted them based on participant feedback, and the English interview transcripts were verified by a second bi-lingual member of the research team to ensure the English version was clear and the participants’ views were adequately represented. The interviews were semi-structured with open-ended questions, which allowed respondents to explore the mentioned topics as they saw fit. Adding more structured interview questions to explore some issues in more detail (e.g., sample collection, distance and subsequent travel time of samples, transport conditions particularly during winter and waste disposal system for cartridges) might have been useful.

In summary, this study identified a number of barriers and enablers of Xpert MTB/RIF implementation in Mongolia that extended beyond purchasing the equipment and installing it locally. The program environment is important for the successful implementation of Xpert MTB/RIF. Our study found that factors affecting implementation centred around awareness of program guidelines, cartridge supply management, local repair options for GeneXpert technology, regular formal training, communication systems and processes for sample collection. Upscaling of Xpert MTB/RIF testing facilities in Mongolia and other low-and middle income countries will lead to implementation of Xpert MTB/RIF in new settings in future, and we believe that the lessons learned from our study can help to facilitate this process.

##  Supplemental Information

10.7717/peerj.3567/supp-1 File S1 Interview Guides

10.7717/peerj.3567/supp-2 Supplemental Information 1 COREQ Checklist

10.7717/peerj.3567/supp-3 Supplemental Information 2 Notified Cases 2011-2016 - referenced in discussion, sourced from NTP annual data

## Additional Information and Declarations

### Data Availability

The following information was supplied regarding data availability: The raw data has been supplied as a Supplemental File.
