# Supplementary material for: Implementation of the Xpert MTB/RIF assay for tuberculosis in Mongolia: a qualitative exploration of barriers and enablers
Source: PeerJ. 2017 Jul 14;5:e3567. doi: 10.7717/peerj.3567 (PMC5511701; doi:10.7717/peerj.3567)
Supplement: File S1 [file peerj-05-3567-s001.pdf]

Interview Questions for laboratory staff

This form is for recording notes on non-verbal communication only. Responses to questions will be recorded using audio recorder and transcribed for analyses.

Date of Interview:
Professional Role (please circle): Laboratory doctor/Laboratory technician/ Laboratory administrative officer
Laboratory (please circle): NRTL/Darkhan-uul/Dornod

Interview introduction (to be read by interviewer):
Thank you for your involvement in our study. As explained on the participant information sheet, the aim of the study is to understand which factors affect implementation of the Xpert MTB/RIF test in Mongolia by asking you about your experiences using GeneXpert. This will help us to understand how processes can be improved, so that we can make better use of the GeneXpert machines.
Your responses will be recorded using an audio recorder and will be later transcribed for analyses. Please provide as much detail as possible in your responses.
We can begin once you have read and signed the consent form.

Framework, Guidelines and Organisational Structures

1. What formal guidance from your managers is in place to help you using GeneXpert? For example, GeneXpert guidelines that help you to understand how to do tests correctly? Do you have written materials that you can consult if you have a question?
    a. When was the first time you received guidelines specific to the use of GeneXpert? What is the content of the guidelines? For example, how to perform GeneXpert tests correctly, how GeneXpert tests should be combined with other tests for TB?
    b. Have you received assistance from international or local experts (outside of your laboratory) on how to use GeneXpert? If yes, what kind of assistance did you receive? Was it helpful? Is this assistance still available to you?

c. Does the assistance you get for performing GeneXpert correctly differ from the assistance you get to perform other TB tests? If yes, how is it different?
d. How has the introduction of GeneXpert affected your workload? If it has increased, what staffing changes have been made?
e. In your opinion, what are the ideal staffing arrangements to perform GeneXpert testing routinely? For example, the number of staff, schedule of shift work, staff qualification, staff knowledge?

Equipment

2. Have you had positive or negative experiences using the GeneXpert machine?
    a. How often do you personally use the GeneXpert machines? How often do other staff use the machines?
    b. What kind of activities do you need to do for GeneXpert machine maintenance? Have you had any problems with machine maintenance? Have some parts of the GeneXpert machine needed replacement? If so, were these parts readily available?
    c. Do you experience machine faults regularly? What is your experience with power supply and temperature faults?
    d. Are there problems with access to cartridges and GeneXpert machine equipment? If yes, please provide details of the problems you have had.
        i. Has short shelf life of cartridges been a challenge?
        ii. Has procurement of cartridges and equipment been a challenge?
        iii. Has transport of cartridges and equipment been a challenge?
    e. Have you experienced or are aware of any other technical difficulties with the GeneXpert machines that we have not already discussed? If yes, how were these addressed? How could they have been avoided?

Training

3. How much training have you received on the use of GeneXpert? What topics did the training cover?
    a. Was the training sufficient for your job requirements? If not, what additional training would have assisted you in meeting your job requirements?

   b. When was your last GeneXpert training session? What materials were provided to you for the purpose of this training?
   c. Is there ongoing training planned specific to GeneXpert?
   d. Do you feel confident handling a GeneXpert machine? If not, what kind of training would you suggest?
   e. Do you receive ongoing training for other diagnostic techniques such as smear microscopy? If yes, how often does this occur?

Communication Systems

4. How are GeneXpert results communicated to doctors and other TB clinics? For example fax, email, hard copy via regular mail, phone call?
   a. Describe the system in place for recording sample information? Does it include information on when samples are received? What testing is undertaken? Final results? Is the system paper based or electronic?
   b. Have you had any issues with patient identification and recording of results? If yes, please describe what happened?
   c. What procedures are in place to check the quality of GeneXpert results? For example, are GeneXpert results regularly collated and compared with culture results for consistency?
   d. What information is *collected* to monitor GeneXpert activities in your laboratory? For example, how many samples are received for GeneXpert testing every month? Is similar information collected for smear microscopy?
      i. In which format is the information collected – paper forms or electronically?
   e. How often is this information *reported*? Is it the same for GeneXpert and smear microscopy?

Diagnostic Algorithms, Case Finding for GeneXpert, Clinical Management

5. Consider the whole testing process from receiving the sample, doing the GeneXpert test and reporting of the results. In practice, have there been any problems with this process?
   a. How much time typically passes between receiving the sample and reporting the results? Is this information regularly reported?

b. Are you aware if GeneXpert testing in Mongolia is used mainly for finding TB cases, testing drug susceptibility or both?
c. Are there concerns about the quality of samples used for GeneXpert testing? If yes, how have these been managed?
d. Have you only processed sputum with GeneXpert or also other body fluids (pleural fluid, urine etc)? Have you processed body tissue from biopsies with GeneXpert?
e. Have there been any problems with GeneXpert requests sent to the laboratory? For example, has there been situations where the information on the request form has been unclear?
f. Have there been many ‘error’ results/invalid results with GeneXpert? If yes, do you know the reason for the invalid results?
g. If you get an error result, what is the usual procedure?
h. What quality control measures are in place for GeneXpert testing and smear microscopy?
i. Have there been many inconsistent results between smear and/or culture and GeneXpert results?
j. How are inconsistent results dealt with?

6. Summary

a. Overall, how does GeneXpert compare to smear microscopy?
b. In the future what changes would you like to see with the implementation of GeneXpert machines in Mongolia?

Interview Questions for TB doctors

This form is for recording notes on non-verbal communication only. Responses to questions will be recorded using audio recorder and transcribed for analyses.

Date of Interview:
Professional role (please circle): Clinical TB Doctor at NCCD/Clinical TB Doctor in district or aimag TB clinic
Provinces (please circle): Ulaanbaatar/Darkhan-uul/Dornod

Interview introduction (to be read by interviewer):
Thank you for your involvement in our study. As explained on the participant information sheet, the aim of the study is to understand which factors affect implementation of the Xpert MTB/RIF test in Mongolia by asking you about your experiences using GeneXpert. This will help us to understand how processes can be improved, so that we can make better use of the GeneXpert machines.
Your responses will be recorded using an audio recorder and will be later transcribed for analyses. Please provide as much detail as possible in your responses.
We can begin once you have read and signed the consent form.

Questions

1. Have you had positive or negative experiences with GeneXpert testing, compared with smear microscopy? Please explain.
    a. Please describe the situation when you would request the GeneXpert test? Do you use it mainly for finding TB cases, drug susceptibility testing or both?
    b. In your opinion, what do you think are the main advantages and disadvantages of using GeneXpert?
    c. When a patient with presumptive pulmonary TB comes to your clinic, which of the following tests do you request – sputum smear examination, chest x-ray, and/or GeneXpert?
        i. In what order do you request these tests? In which patients would you use GeneXpert as the first test?

d. Are you aware of any guidelines* about which patients to test with GeneXpert? If yes, would you mind summarising your understanding of the guidelines and how this impacts on your practice?
e. What do you do if you get a report of rifampicin resistance using GeneXpert? What treatment do you give the patient if he/she is:
    i. new (currently not on treatment)?
    ii. currently on first line treatment?
f. Have you been in a situation where you wanted to start a patient on MDR-TB treatment based on rifampicin resistance diagnosed using GeneXpert, but the treatment was not available? If yes, does that happen frequently? Why?
g. On average, how long does it take to receive the laboratory results after sending the sample to the laboratory? How do you receive the results - paper, email, phone call?
h. Once you have a positive TB result, how long does it take for that patient to begin treatment? Is there any difference between treatment start/delay between patients diagnosed on sputum smear and those diagnosed on GeneXpert?
i. Have you experienced any problems when requesting a GeneXpert test?

2. Summary

a. Overall, how does GeneXpert compare to smear microscopy?
b. In the future what changes would you like to see with the implementation of GeneXpert machines in Mongolia?
