# Supplementary material for: Implementation of the Xpert MTB/RIF assay for tuberculosis in Mongolia: a qualitative exploration of barriers and enablers
Source: PeerJ. 2017 Jul 14;5:e3567. doi: 10.7717/peerj.3567 (PMC5511701; doi:10.7717/peerj.3567)
Supplement: Supplemental Information 1 [file peerj-05-3567-s002.docx]

**Implementation of the Xpert MTB/RIF assay for tuberculosis in Mongolia: a qualitative exploration of barriers and enablers –**

**Consolidated criteria for reporting qualitative studies (COREQ): 32-item checklist**

| No | Item | Guide questions/description | Response |
| --- | --- | --- | --- |
| Domain 1: Research team and reflexivity |  |  |  |
| Personal Characteristics |  |  |  |
| 1. | Interviewer/facilitator | Which author/s conducted the interview or focus group? | Gantungalag Ganbaatar and Munkhjargal Dorjravdan |
| 2. | Credentials | What were the researcher's credentials? *E.g. PhD, MD* | Nicole Rendell – MPH  Solongo Bekhbat - MD  Gantungalag Ganbaatar - Epidemiologist  Munkhjargal Dorjravdan – MD  Madhukar Pai - MD, PhD  Claudia C Dobler – MD, MBBS, MD, PhD, FRACP |
| 3. | Occupation | What was their occupation at the time of the study? | Nicole Rendell – Monitoring and Evaluation Advisor, National Tuberculosis Program Mongolia  Solongo Bekhbat – Executive Director, Mongolian Anti-Tuberculosis Association  Gantungalag Ganbaatar – Epidemiologist, National Tuberculosis Program Mongolia  Munkhjargal Dorjravdan – TB doctor specialising in MDR-TB, National Tuberculosis Program Mongolia  Madhukar Pai - Canada Research Chair in Epidemiology & Global Health  Director, McGill Global Health Programs  Associate Director, McGill International TB Centre, McGill University, Montreal, Canada  Claudia C Dobler - Consultant Respiratory Physician, Conjoint Senior Lecturer UNSW and UWS, Dept of Respiratory Medicine, NHMRC TRIP (translating research into practice) fellow, Woolcock Institute of Medical Research, Sydney, Australia |
| 4. | Gender | Was the researcher male or female? | Nicole Rendell – female  Solongo Bekhbat – female  Gantungalag Ganbaatar – female  Munkhjargal Dorjravdan – female  Madhukar Pai – male  Claudia C Dobler – female |
| 5. | Experience and training | What experience or training did the researcher have? | Nicole Rendell – post-graduate level  Solongo Bekhbat – post-graduate level  Gantungalag Ganbaatar – post-graduate level  Munkhjargal Dorjravdan – post-graduate level  Madhukar Pai – extensive experience  Claudia C Dobler – extensive experience |
| Relationship with participants |  |  |  |
| 6. | Relationship established | Was a relationship established prior to study commencement? | Nicole Rendell – No  Solongo Bekhbat – No  Gantungalag Ganbaatar – colleague  Munkhjargal Dorjravdan – colleague  Madhukar Pai – No  Claudia C Dobler – No |
| 7. | Participant knowledge of the interviewer | What did the participants know about the researcher? e*.g. personal goals, reasons for doing the research* | Gantungalag Ganbaatar – knowledge of general personal information consistent with collegiate relationships, role within the organisation and context of the research.  Munkhjargal Dorjravdan – knowledge of general personal information consistent with collegiate relationships, role within the organisation and context of the research. |
| 8. | Interviewer characteristics | What characteristics were reported about the interviewer/facilitator? e.g. *Bias, assumptions, reasons and interests in the research topic* | Gantungalag Ganbaatar – no bias was reported and interests in the research topic were well known.  Munkhjargal Dorjravdan – no bias was reported and interests in the research topic were well known. |
| Domain 2: study design |  |  |  |
| Theoretical framework |  |  |  |
| 9. | Methodological orientation and Theory | What methodological orientation was stated to underpin the study? *e.g. grounded theory, discourse analysis, ethnography, phenomenology, content analysis* | Inductive-deductive approach. |
| Participant selection |  |  |  |
| 10. | Sampling | How were participants selected? *e.g. purposive, convenience, consecutive, snowball* | Purposive. |
| 11. | Method of approach | How were participants approached? e*.g. face-to-face, telephone, mail, email* | Face-to-face and over the phone. |
| 12. | Sample size | How many participants were in the study? | 24 |
| 13. | Non-participation | How many people refused to participate or dropped out? Reasons? | None. |
| Setting |  |  |  |
| 14. | Setting of data collection | Where was the data collected? e*.g. home, clinic, workplace* | In one of the meeting rooms of the National Tuberculosis Program office or over the phone while the interview was based in the office. |
| 15. | Presence of non-participants | Was anyone else present besides the participants and researchers? | No. |
| 16. | Description of sample | What are the important characteristics of the sample? *e.g. demographic data, date* | Use of Xpert MTB/RIF as part of their work as a TB doctor or laboratory staff. |
| Data collection |  |  |  |
| 17. | Interview guide | Were questions, prompts, guides provided by the authors? Was it pilot tested? | Yes prompts were provided but the interviewers but the participants did not have visibility of the guides. Pretesting of the interview guides was undertaken. |
| 18. | Repeat interviews | Were repeat interviews carried out? If yes, how many? | No. |
| 19. | Audio/visual recording | Did the research use audio or visual recording to collect the data? | Audio recorder. |
| 20. | Field notes | Were field notes made during and/or after the interview or focus group? | No. |
| 21. | Duration | What was the duration of the interviews or focus group? | It varied between participants but generally between 20-30 minutes. |
| 22. | Data saturation | Was data saturation discussed? | Yes, among researchers. |
| 23. | Transcripts returned | Were transcripts returned to participants for comment and/or correction? | No. |
| Domain 3: analysis and findingsz |  |  |  |
| Data analysis |  |  |  |
| 24. | Number of data coders | How many data coders coded the data? | Two. |
| 25. | Description of the coding tree | Did authors provide a description of the coding tree? | No not a coding tree but a framework with definitions and examples was used to facilitate consistent coding between the authors. |
| 26. | Derivation of themes | Were themes identified in advance or derived from the data? | High level themes were identified in advance and subthemes were derived from the data. |
| 27. | Software | What software, if applicable, was used to manage the data? | QSR NVivo version 10 (QSR International Pty Ltd, Doncaster, Victoria, Australia) |
| 28. | Participant checking | Did participants provide feedback on the findings? | No but the translation of the transcripts were verified by a second researcher to ensure accuracy. |
| Reporting |  |  |  |
| 29. | Quotations presented | Were participant quotations presented to illustrate the themes / findings? Was each quotation identified? e*.g. participant number* | Earlier drafts had taken this approach and then were removed as part of the editing process due to concerns about length of the paper. |
| 30. | Data and findings consistent | Was there consistency between the data presented and the findings? | Yes. |
| 31. | Clarity of major themes | Were major themes clearly presented in the findings? | Yes. |
| 32. | Clarity of minor themes | Is there a description of diverse cases or discussion of minor themes? | Yes. |
