# Supplementary material for: Implementation of the Xpert MTB/RIF assay for tuberculosis in Mongolia: a qualitative exploration of barriers and enablers
Source: PeerJ. 2017 Jul 14;5:e3567. doi: 10.7717/peerj.3567 (PMC5511701; doi:10.7717/peerj.3567)
Supplement: Supplemental Information 2 [file peerj-05-3567-s003.docx]

**Number of notified cases, Ulaanbaatar, Darkhan and Dornod, 2011-2016**

| **Year** | **Drug susceptible TB** | **Drug resistant TB** | **Total** |
| --- | --- | --- | --- |
| 2011 | 2626 | 128 | 2754 |
| 2012 | 2647 | 136 | 2783 |
| 2013 | 2784 | 139 | 2923 |
| 2014 | 2870 | 114 | 2984 |
| 2015 | 2910 | 119 | 3029 |
| 2016 | 3186 | 156 | 3342 |

Source: NTP annual data
